# Structural equation model based on salutogenesis theory for evaluating factors affecting health-related quality of life in adolescents with moyamoya disease

**DOI:** 10.1038/s41598-022-24825-y

**Published:** 2022-11-27

**Authors:** Won-oak Oh, Insun Yeom, Sung-Hyun Lim

**Affiliations:** 1 Korea University College of Nursing, 145 Anam-ro, Seongbuk-gu, Seoul, Republic of Korea; 2 Brain Korea 21 FOUR Project, College of Nursing, Yonsei University, Seoul, Republic of Korea

**Keywords:** Neuroscience, Neurology

## Abstract

Moyamoya disease is a cerebrovascular disorder and a significant chronic health concern requiring regular monitoring to control the disease and its related complications. We examined a hypothetical model by integrating the concepts of a structural health-related quality-of-life model based on the salutogenesis theory, and to identify how social support, sense of coherence, and stress contribute to health behaviors, subjective health status, and quality of life in adolescents with moyamoya disease among 239 adolescents in Korea. A structural equation model was used to analyze the data. The fitness of the hypothetical model with the salutogenesis theory was satisfactory, showing that the goodness-of-fit index = 0.91, adjusted goodness-to-fit index = 0.90, comparative fit index = 0.92, normed fit index = 0.91, incremental fit index = 0.91, standardized root mean squared residual = 0.04, root mean square error of approximation = 0.07, parsimony normed fit index = 0.61, parsimony goodness of fit index = 0.51. The model explained 68.9% of quality of life. Health behavior (β = −0.173, p = 0.467) and stress (β = −0.557, p < 0.001) had significant direct and total effects on quality of life. Sense of coherence had a significant direct (β = 0.371, p = 0.003), indirect (β = 0.220, p = 0.013), and total (β = 0.590, p < 0.001) effect on quality of life. This study found that sense of coherence was significant factors contributing to lower stress, improved health status, and quality of life in adolescents with moyamoya disease. To improve the quality of life for adolescents with moyamoya disease, comprehensive nursing interventions need to be developed and applied.

## Introduction

With the development of modern medical technology, research on rare and incurable diseases is conducted more regularly. For this reason, we can now monitor rare, incurable, and chronic diseases on a daily basis. Moyamoya disease, a rare and incurable disease, is a primary cause of strokes or transient ischemic attacks in adolescents^1^. The prevalence of moyamoya disease is reported to be 3.16 per 100,000 people, and the incidence rate is reported to be 0.5 per 100,000 people per year^2^. The highest incidence rates are among people aged 5–15 and 30–49 years. Incidences of moyamoya disease in adolescents have increased in recent years, requiring continuous management despite surgical treatments^3^.

Transient ischemic attacks affect approximately 70% of adolescents with moyamoya disease and symptoms such as headaches, hemiparesis, and seizures may occur temporarily or permanently^4–6^. Decreased brain perfusion can cause cognitive impairments, intellectual decline, and mental retardation^3^.

To prevent symptoms that cause severe sequelae, reducing risk factors and managing symptom triggers is essential. In terms of disease management, it is important to identify the health behaviors required for patients to manage moyamoya disease and the degree to which they must be implemented. Moyamoya disease can easily cause cerebral vascular hypo circulation-related symptoms even with minor changes in blood flow. It can also cause transient cerebral ischemic attacks in stressful situations^7^; therefore, stress management is required^2,8^. The health issues and symptoms experienced by adolescents with moyamoya disease are often stress-related^9^. Therefore, it is necessary to understand the relationship between stress and health in adolescents with moyamoya disease to prevent the exacerbation of these problems and provide age-appropriate stress and health-management interventions.

Few studies have explored the promotion of healthy behaviors among adolescents with moyamoya disease in-depth. Therefore, to promote the health of these adolescents, it is important first to understand the typical health status of moyamoya disease patients and identify factors affecting their health and quality of life.

In the case of adolescent patients with moyamoya disease, although the most prevalent age group is the age in which social activities become more sophisticated, they experience reduced independence and weakened autonomy due to changes in activity due to the disease, which leads to a decrease in quality of life^10^. Quality of life refers to subjective well-being for life^11^, and chronic disease patients experience a chaotic reality due to changes due to disease, and stress is caused by physical and psychological factors. This leads to a decrease in the quality of life, and at this time, it is important to provide appropriate support and intervention in order to adapt to a new life^12^. A study on children with moyamoya also showed a high psychological burden and a marked decrease in quality of life^10^.

According to the Bangkok Charter adopted at the Sixth Global Conference on Health Promotion, "Health promotion is the process by which a person develops the ability to control his or her health and its determinants, thereby becoming healthier"^13^. This means that an individual's organizational and social skills should be nurtured, and appropriate resources provided to identify health problems and find the best solution for them. Therefore, it is necessary to provide a fresh perspective and construct a new concept of health status for adolescents with moyamoya disease.

The salutogenesis theory emphasizes the importance of individual ability and resources to nurture health rather than reducing external stressors. Sense of coherence has been suggested as a critical factor in controlling stress^14^.

A previous study applying the salutogenesis theory reported that stress symptoms were reduced^15^, health behaviors were well performed, and overall health was good^16^. In the group with a high sense of coherence and social support among adolescents, stress perception was lowered by leading stressful situations in the direction of comfort^17–19^. On the other hand, when adolescents with low sense of coherence were faced with a stressor, they reported high stress levels because they could not adequately resolve tension^20,21^. Also, in the case of adolescents with chronic diseases, the higher the sense of coherence, the better the health behavior^22,23^. In another report, it was confirmed that the higher the level of integration, the more successful the stressors were resolved, and thus, ultimately, it was confirmed that a positive result was given to an individual's quality of life^24,25^.

The salutogenesis theory was integrated with this study, and it seemed a suitable model for promoting health in adolescents with moyamoya disease. This theory emphasizes the individual's ability to use sense of coherence and manage within a stressful environment. It explains the problem-solving process in greater detail so that the individual may more easily adopt health-promoting behaviors^14^.

Adolescents with moyamoya disease are more stressed than other adolescents due to their disease and other developmental challenges, triggering adverse health conditions and symptoms. Sense of coherence and social support have been shown to assist in recovery and promote health by improving an individual's ability to cope with stress as reported in studies of adolescents with other chronic diseases^26^. These results indicate that it is necessary to include social support in the model.

Therefore, this study, which builds a hypothetical model based on salutogenesis theory^14^ and literature review^17–25^, examines the effects of factors such as sense of coherence, stress, social support, health behaviors, and health status on quality of life. The intention was to check it holistically and multi-dimensionally. The results of this study will provide basic data for the development of practical and effective nursing interventions to improve the quality of life of adolescents with moyamoya disease.

## Conceptual framework and hypothetical model

**Figure 1:**
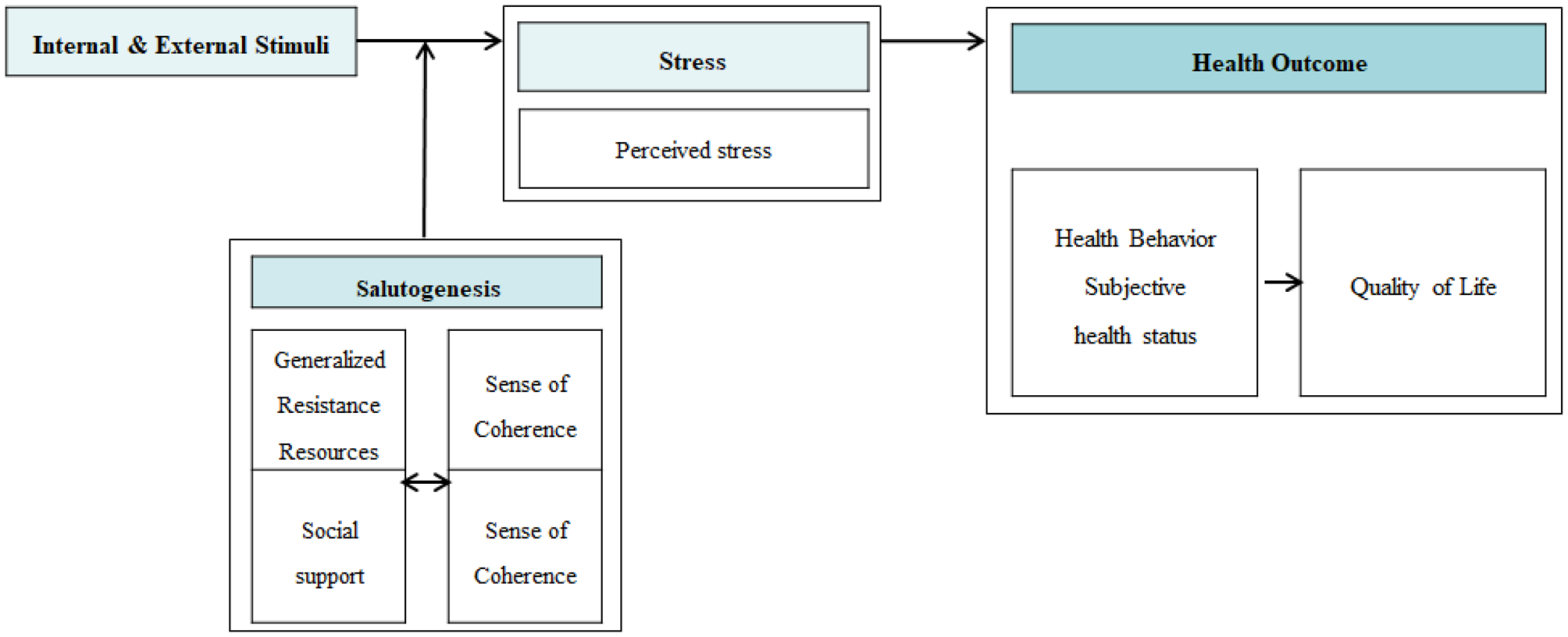
Theoretical framework based on the salutogenesis theory. x1 = social support (parent), x2 = social support (teacher + close friend), x3 = sense of coherence (meaningfulness), x4 = sense of coherence (manageability), x5 = sense of coherence (comprehensibility), y1 = stress (behavior), y2 = stress (physical), y3 = stress (emotion), y4 = healthy behavior (treatment instructions, health coping behavior, health promotion acts), y5 = self-management confidence, y6 = quality of life (physical), y7 = quality of life (emotional), y8 = quality of life (interpersonal relationship + school).

This study constructed a conceptual framework through literature review based on Antonovsky's salutogenesis theory (Fig. 1). In the salutogenesis theory, humans are open systems that actively interact with the environment. Interaction with the external environment can be a potential source of disease in stressful situations where people are ill, but it can also be a resource to promote health. Despite the inevitable stress and illness experienced in an individual's life, the theory explains that recovery is possible by actively overcoming stress through the internal abilities and resources of the individual. In other words, essential factors of the health promotion process are generalized resistance resources and sense of coherence^14^. In this study, when social support and sense of coherence are high, stress is low, which leads to better health behavior performance and subjective health status, which leads to better quality of life as the final health outcome. Based on this, social support^17^ and sense of coherence^17–19^ affect stress, stress affects health behavior performance^22,23^ and subjective health status^23^, and finally quality of life^24,25^ is established. Therefore, we hypothesize that social support and sense of coherence affect individual stress and health behaviors, affecting subjective health status, and quality of life.

Based on these path settings, a hypothetical path was constructed, as shown in Fig. 1.

## Methods

### Design

This was a single center, cross-sectional study based on the Salutogenesis theory. This study used a structural equation model to suggest and verify a hypothetical model for the factors influencing health-related quality of life in adolescents with moyamoya disease, based on the salutogenesis theory^14,27^.

### Participants

The sample included 239 adolescents aged 13–18 years with moyamoya disease in the moyamoya clinic at Severance Hospital in South Korea. The inclusion criteria were those who had been diagnosed with moyamoya disease (Korea Standard Classification of Disease: I67.5) by a neurosurgeon at least 1 month before participation in the study, had no other diseases, were able to communicate, understood the purpose of the study, and gave permission to participate in the study. In addition, the exclusion criteria for adolescents with moyamoya disease were a history of mental illness or difficulty in participating (e.g., hearing or visual impairment). All participants confirmed their understanding of the purpose of the study and agreed to participate voluntarily through online written consent. Written informed consent was obtained from parents of participants 16 years of age or lower.

In general, the size of the sample required to verify the structural equation model is presented as a critical value of about 200 samples^28^. Based on this recommendation, the survey was distributed to 250 participants, allowing for a 20% dropout rate. The final number of participants was 239, which excluded 11 participants who had did not sufficiently fill out the questionnaire.

### Data collection

Data were collected after approval by the Severance Hospital Institutional Review Board (4-2017-0884). All procedures performed in this study were in accordance with the ethical standards of the institutional or national research committee. Data were collected face-to-face between January and October of 2018. One researcher and a neurosurgery outpatient nurse collected questionnaire data through 1:1 interview when a patient visited the outpatient clinic for examination or examination. When subjects and their parents consented to participate in the study, they were asked to sign a consent form and fill out a questionnaire. Participants were assured of the anonymity and confidentiality of their responses and that any data collected would be used for research purposes only. They were told that they could withdraw from the study at any time. Those who agreed to participate answered the questions. After the survey, the participants each received a gift from the researcher for completing the survey.

### Measurement instruments

#### Social support

For social support, the Child and Adolescent Social Support Scale (CASSS) developed by Malecki and Demaray^29^ was translated into Korean by this researcher and used after the translation-reverse translation process was performed.

A translated child and adolescent social support scale was completed by each participant. The self-reported questionnaire consisted of 48 items and was scored on a 6-point Likert scale. Support was assessed on four subscales: support from parents (12 items), support from teachers (12 items), support from classmates (12 items), and support from close friends (12 items).

Each question is scored on a 6-point Likert scale with 1 point for ‘not at all’ and 6 points for ‘always’. Higher scores indicated more social support in each area. Cronbach's alpha of the scale at the time of development was 0.96, and in this study, Cronbach's alpha was 0.93.

#### Sense of coherence

The sense of coherence scale^14^ was used to measure each participant’s sense of coherence. The author translated the scaleinto Korean and then re-translated it back to English to ensure accuracy. The scale was developed using two scales; the original scale measured 29 items, and the shortened scalel measured 13 items. Because the reliability and validity of the shortened scale were verified, this study used the shortened scale for convenience.

The self-reported questionnaire consisted of 13 items and was scored on a 7-point scale, making the score range 13–91. Three sections were measured: comprehensibility (5 items), manageability (4 items), and meaningfulness (4 items). Higher scores indicated a higher sense of coherence. Cronbach's alpha of the scale at the time of development was 0.87, and in this study, Cronbach's alpha was 0.88.

#### Health behavior

The moyamoya health behavior scale (developed by the author) was used to determine the health behavior of adolescents with moyamoya disease^6^. This scale consists of health behaviors that adolescent patients with moyamoya disease should perform as health behaviors related to the implementation of moyamoya disease treatment instructions, health coping behaviors and health promotion. The self-reported questionnaire consisted of 12 items and was scored on a 5-point scale; therefore, the score range was 12–60. Three sections were measured: treatment instructions (4 items), health promotion acts (4 items), and health coping behavior (4 items). Higher scores indicated better health behaviors related to moyamoya disease. When the moyamoya health behavior scale was developed, Cronbach's α was 0.86; in this study, it was 0.87.

#### Stress

Stress was assessed using the stress scale for adolescents^30^. The scale consists of three subscales measuring physical (9 questions), behavioral (9 questions), and emotional (9 questions) stress. All items are scored on a 4-point asymmetrical rating scale, ranging from 1 (not at all) to 4 (a lot). There were 27 questions, and the higher the score for each sub-factor, the more stress was experienced. At the time of the scale's development, Cronbach's α was 0.82; in this study, it was 0.95.

#### Subjective health status

Subjective health status is to evaluate an individual's health inclusive of physical, physiological, psychological, and social aspects. In this study, subjective health status was a subjective evaluation of the health perceived by adolescents with moyamoya disease regarding their current health status. Subjective health status was a subjective evaluation of the perceived health of the participants. This study consisted of a 5-point simple-question Likert scale, ranging from 1 point (very bad) to 5 points (very good).

#### Quality of life

Quality of life assesses subjective satisfaction with the academic functions and physical, mental, and social well-being that one perceives in one's life^31^. The quality of life was translated and standardized by Kook and Vanri^32^ of the 4th edition (Pediatric Quality of Life InventoryTM4.0 Generic Core Scales) developed by Varni et al.^18^. It was measured using the Korean Translation of the Pediatric Quality of Life Inventory™ 4.0 Generic Core Scales. This scale consists of 23 items in total, consisting of 8 items on physical function, 5 items on emotional function, 5 items on interpersonal function, and 5 items on school function. All the items were composed of items opposite to positive quality of life, such as 'I am sad'. Therefore, for each item, 'never (100 points)', 'rarely (75 points)', 'sometimes (50 points)', 'often (25 points)', 'very often (0 points)' The scale developer suggested to convert it to. The higher the measured score, the higher the quality of life. In the standardization study, Cronbach's alpha was 0.90 for all items of the scale for reporting children. Cronbach's alpha in this study was 0.91.

### Data analysis

We analyzed the data using IBM SPSS^®^ version 26.0 and AMOS version 25.0. The general characteristics and study variables were summarized using descriptive statistics. Factor loading, average variance extraction, and critical ratio were utilized for the convergent validity of the sample. The discriminant validity between variables was determined using the correlation coefficient (*r*) and the average variance extracted; statistical data on skewness and kurtosis were obtained for the normality of the samples (< **± **3)^33,34^. Multicollinearity between variables was determined using variance inflation factor and tolerance. Absolute values of the correlation coefficients were evaluated (< 0.85), confirming that the discriminant validity between factors was secured^28^. A confirmatory factor analysis was performed on the model to evaluate whether the measurement for the latent variable was executed correctly. The goodness-of-fit of the measurement model, hypothetical model, and modified model was evaluated using χ^2^ (p < 0.001), χ^2^/df (< 3.0), adjusted goodness-of-fit index (0.9–1), goodness-of-fit index (0.9–1), comparative fit index (0.9–1), root mean square error of approximation (≤ 0.08), standardized root mean squared residual (≤ 0.05), parsimony normed fit index (> 0.5), and parsimony goodness of fit index (> 0.5).

We used standard regression weights, critical ratios, *p* values, and squared multiple correlations to verify the statistical significance of the modified model. The bootstrap maximum estimator was used 10,000 times within the 95% confidence interval to test the significance of the modified model's direct, indirect, and total effects.

The datasets used and/or analyzed during the current study are available from the corresponding author on reasonable request.

### Ethical considerations

After receiving approval from the IRB of the investigator’s affiliated hospital (4-2017-0884), subjects were recruited and data were collected. Patients and patient's parent interested in the study were provided with detailed paper instructions. Subjects who voluntarily indicated their intention to participate in the study provided written informed consent.

## Results

### Participants' general characteristics and key variables related to study

The participants who responded were 49% male and 51% female, with an average age of 14.9 (**± **2.1). Of the study participants, 93.7% were confirmed as having moyamoya disease during a hospital visit for related symptoms such as convulsions or ischemic attack, 2.5% were found to have the disease during a routine medical examination, and 3.8% were discovered to have the disease during an examination related to another disease. The duration since diagnosis was less than one year (16.7%), 1 to 3 years (31.8%), and > 3 years (51.5%). Finally, 20.9% had been educated about the disease before the study (Table 1).

**Table 1:** Participant characteristics (N = 239).

Variables	Categories	n (%)	Mean ± SD
Gender	Male	117 (49.0)	
	Female	122 (51.0)	
Age (years)	13–15	167 (69.9)	13.5 (± 0.9)
	16–18	72 (30.1)	17.1 (± 1.1)
Confidence of self-management	Cannot do it at all	3 (1.3)	
	Can do well	20 (8.4)	
	Usually	132 (55.2)	
	Can do well	64 (26.8)	
	Can do very well	20 (8.4)	
Diagnosed path^a^	Clinical symptom	224 (93.7)	
	Medical examination	6 (2.5)	
	Detected during examination of another disease	9 (3.8)	
Period since diagnosis (months)	0–12	40 (16.7)	7.9 (± 4.1)
	13–36	76 (31.8)	26.1 (± 9.6)
	≥ 37	123 (51.5)	52.9 (± 12.7)
Experience in receiving disease information and disease education	Yes	50 (20.9)	
	No	189 (79.1)	

^a^Confirmed as having moyamoya disease.

The average scores for the participants’ social support was 163.6 (± 32.11), sense of coherence was 61.78 (± 12.04), stress was 43.71 (± 14.98), moyamoya disease health behavior was 41.13 (± 9.19), and quality of life was 92.43 (± 14.99).

Before the structural model analysis, skewness and kurtosis were investigated to check whether the variables were normally distributed. Results showed that skewness was confirmed as −2.82 to 2.73, and kurtosis as −1.87 to 2.61 (Table 2).

**Table 2:** Descriptive statistics and confirmatory factor analyses of measured variables (N = 239).

Variables	Number of question	Mean (± SD)	Range	Skewness	Kurtosis
**Social support**					
Total	36	163.60 (± 32.11)	65–216	−1.03	−1.69
Parent (1–12)	12	54.42 (± 12.65)	14–72	−2.76	−1.79
Teacher (13–24) + close friend (25–36)	24	109.18 (± 22.76)	51–144	−1.74	−1.78
**Sense of coherence**					
Total	13	61.78 (± 12.04)	27–90	1.77	−1.64
Comprehensibility (2, 6, 8, 9, 11)	5	23.31 (± 5.21)	11–35	1.67	−1.87
Manageability (3, 5, 10, 13)	4	19.35 (± 4.17)	8–28	0.80	−1.87
Meaningfulness (1, 4, 7, 12)	4	19.12 (± 4.06)	6–28	0.50	−0.22
**Stress**					
Total	27	43.71 (± 14.98)	16–103	2.53	2.71
Physical (15–27)	13	20.42 (± 7.46)	0–51	2.73	2.29
Behavior (1–5, 8)	6	10.9 3 (± 3.88)	6–23	2.30	2.12
Emotion (6, 7, 9–14)	8	12.36 (± 5.18)	6–32	2.53	2.55
**Health behavior**					
Treatment instructions (1–4) + health coping behavior (5–8) + health promotion acts (9–12)	12	41.13 (± 9.19)	15–60	−1.21	−0.64
**Quality of life**					
Total	23	92.43 (± 14.99)	45–115	−24.00	0.98
Physical function (1–8)	8	31.43 (± 5.82)	13–40	−2.43	−1.58
Emotional function (9–13)	5	20.09 (± 4.22)	5–25	−2.13	0.61
Interpersonal skills (14–18) + school function (19–23)	10	40.91 (± 7)	18–50	−2.82	1.54

Confirmatory factor analysis was performed to evaluate the validity of the constituent factors. Table 3 present confirmatory factor analysis results to evaluate the validity of the constituent factors. In the test for the constituent factors, all fitness indices met the criteria (standardized factor loading = 0.73–0.94 (0.5–0.9), concept reliability value = 0.76–0.88 (> 0.7), average variance extracted value = 0.62–0.72 (> 0.5), correlation coefficient between the latent variables of the measurement model = 0.61–0.75 (< 0.85). CFA showed that the discriminant validity between factors was secured.

**Table 3:** Correlation among the observed variables.

	x1	x2	x3	x4	x5	y1	y2	y3	y6	y7	y8
x1	1.000										
x2	0.614***	1.000									
x3	0.451***	0.509***	1.000								
x4	0.461***	0.474***	0.751***	1.000							
x5	0.489***	0.583***	0.705***	0.630***	1.000						
y1	−0.147*	−0.098	−0.229**	−0.172**	−0.205**	1.000					
y2	−0.119	−0.172**	−0.207**	−0.167*	−0.206**	0.671***	1.000				
y3	−0.097	−0.218**	−0.303***	−0.230***	−0.286***	0.764***	0.719***	1.000			
y6	0.174**	0.274***	0.414***	0.282***	0.463***	−0.387***	−0.387***	−0.443***	1.000		
y7	0.196**	0.375***	0.511***	0.399***	0.458***	−0.452**	−0.583***	−0.659***	0.639***	1.000	
y8	0.293***	0.506***	0.425***	0.376***	0.481***	−0.268***	−0.312***	−0.428***	0.683***	0.626***	1.000

x1 = social support (parent), x2 = social support (teacher + close friend), x3 = sense of coherence (meaningfulness), x4 = sense of coherence (manageability), x5 = sense of coherence (comprehensibility), y1 = stress (behavior), y2 = stress (physical), y3 = stress (emotion), y6 = quality of life (physical), y7 = quality of life (emotional), y8 = quality of life (interpersonal + school).

p < 0.05: *, p < 0.01: **, p < 0.001: ***.

### Analysis of structural model

#### Verification of fit for the hypothetical and revised models

The reliability and validity of each latent variable observed was confirmed through confirmatory factor analysis to verify the model’s fit in this study. The fit of the measurement model composed of the confirmed latent variables was verified.

The fit of the initial model of this study was χ^2^ = 469.359 (< 0.001), χ^2^/dt = 4.47, goodness-of-fit index = 0.814, adjusted goodness-to-fit index = 0.729, comparative fit index = 0.854, normed fit index = 0.821, incremental fit index = 0.855, standardized root mean squared residual = 0.075, root mean square error of approximation = 0.121. This indicates that correction is necessary to improve the fit of the model.

During confirmatory factor analysis, it was confirmed that Cronbach's α coefficient for moyamoya health behavior was low when divided by each observation variable (Cronbach's α = 0.578–788). Upon confirming that Cronbach's α coefficient improved when the observed variables were grouped (Cronbach's α = 0.856), the observed variables of the moyamoya health behavior (treatment instructions, health coping behavior, and health promotion acts) were linked and modified.

The fit of the model could be improved when the model was modified by linking the variables with high correlation coefficients between observed variables (and with a correction index of 10 or more) in stages^35^. Based on this, the model was modified by linking the covariance of the observed variable (teacher–close friend) in the corresponding social support variable with the observed variable (interpersonal relationship function–school function) in the quality-of-life variable.

The final model fit index of the hypothetical model in this study was χ^2^ = 211.45 (< 0.001), χ^2^/dt = 4.07,goodness-of-fit index = 0.91, adjusted goodness-to-fit index = 0.90, comparative fit index = 0.92, normed fit index = 0.91, incremental fit index = 0.91, standardized root mean squared residual = 0.04, root mean square error of approximation = 0.07, parsimony normed fit index = 0.61, parsimony goodness of fit index = 0.51. This confirms that the fitness index increased.

The fit of comparative fit index, incremental fit index, and parsimonious fit index confirms that the optimal model criterion is satisfied. Based on the goodness-of-fit index, normed fit index, standardized root mean squared residual values, and parsimony goodness of fit index the hypothetical model for the health of adolescents with moyamoya disease based on the salutogenesis theory was judged to be suitable.

#### Estimating the path coefficient for a hypothetical model

As a result of the hypothetical model analysis, seven out of fourteen pathways in the hypothetical structural model were statistically significant as seen in Table 4 below.

**Table 4:** Standardized estimates, standardized direct, indirect and total effects of the modified mode (n = 239).

Endogenous variables	Explanatory variable	Estimate	Standardized estimates (β)	S.E	CR (p)	SMC (R2)	Standardized direct effects	Standardized indirect effects	Standardized total effects
Stress	Social support	0.034	0.052	0.085	0.402	0.113	0.052 (0.687)	–	0.052 (0.687)
	Sense of coherence	−0.684	−0.373	0.231	−2.958**		0.373 (0.004**)	–	−0.373 (0.004**)
Health behavior	Social support	0.321	0.325	0.115	2.788**	0.274	0.325 (0.006**)	−0.007 (0.568)	0.318 (0.005**)
	Sense of coherence	0.494	0.178	0.317	1.560		0.178 (0.165)	0.049 (0.027*)	0.227 (0.050)
	Stress	−0.198	−0.131	0.097	−2.038*		−0.131 (0.043*)	–	−0.131 (0.043*)
Subjective health status	Health behavior	0.003	0.032	0.006	0.474	0.295	0.032 (0.628)	–	0.032 (0.628)
	Stress	0.002	0.015	0.008	0.242		0.015 (0.828)	−0.004 (0.487)	0.011 (0.879)
	Social support	0.007	0.084	0.010	0.723		0.084 (0.486)	0.011 (0.567)	0.094 (0.441)
	Sense of coherence	0.112	0.467	0.027	4.146***		0.467 (< .001***)	0.001 (0.879)	0.468 (< .001***)
Quality of life	Subjective Health status	0.613	0.108	0.331	1.855	0.689	0.108 (0.056)	–	0.108 (0.056)
	Health behavior	−0.085	−0.173	0.029	−2.968**		−0.173 (0.009**)	0.003 (0.450)	−0.169 (0.010*)
	Stress	−0.414	−0.557	0.049	−8.399***		−0.557 (< .001***)	0.024 (0.053)	−0.534 (< .001***)
	Social support	0.066	0.137	0.048	1.370		0.137 (0.271)	−0.074 (0.339)	0.063 (0.689)
	Sense of coherence	0.504	0.371	0.141	3.575***		0.371 (0.003**)	0.220 (0.014*)	0.590 (< .001***)

*CR* critical ratio, SMC squared multiple correlations.

*p < 0.05, **p < 0.01, ***p < 0.001.

Social support was an insignificant path for stress (β = 0.052, *p* = 0.688) and a significant path for sense of coherence (β = − 0.373, *p* = 0.003). The explanatory power of the variables influencing the stress variable was 1.13%.

Social support for moyamoya health behavior (β = 0.325, *p* = 0.005) and stress (β = −0.131, *p* = 0.042) were found to be significant pathways, while sense of coherence (β = 0.178, *p* = 0.119) was an insignificant path. The explanatory power of the variables affecting the moyamoya health behavior was 27.4%.

Moyamoya disease health behavior (β = 0.032, *p* = 0.636), stress (β = 0.015, *p* = 0.809), and social support (β = 0.084, *p* = 0.469) were not significant pathways for subjective health status while sense of coherence (β = 0.467, *p* < 0.001) was a significant pathway. The explanatory power of the variables affecting subjective health status was 29.5%. Subjective health status (β = 0.108, *p* = 0.064) and social support (β = 0.137, *p* = 0.171) for quality of life were not found to be insignificant.

**Figure 2:**
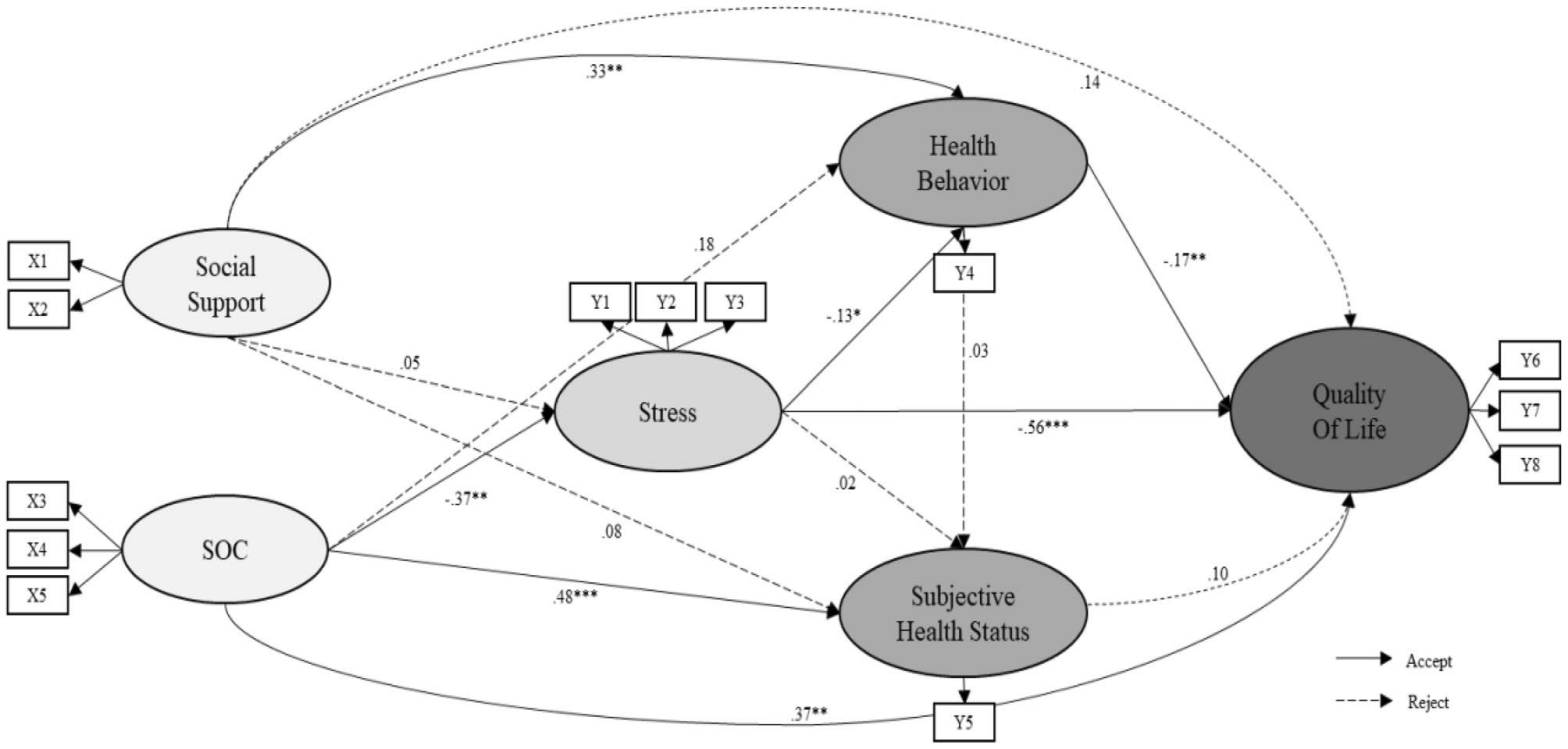
Path diagram for the hypothetical structural equation model.

Moyamoya health behavior (β = −0.173, *p* = 0.003), stress (β = −0.557, *p* < 0.001), and sense of coherence (β = 0.371, *p* < 0.001) were significant pathways for quality of life. The explanatory power of the variables affecting quality of life was 68.9%. The path of the hypothetical model for the above results is shown in Fig. 2.

#### Effects of the modified model

Table 4 shows the direct, indirect, and total effects of the endogenous variables in the modified model. Sense of coherence had a significant direct effect (β = −0.373, *p* = 0.004) on the stress of moyamoya disease adolescents. Social support directly influenced health behavior (β = 0.321, p < 0.01) and subjective health status (β = 0.112, p < 0.01). Social support (β = 0.325, *p* = 0.006) and stress (β = −0.131, *p* = 0.043) had significant direct and total effects on the health behaviors of adolescents with moyamoya disease, and sense of coherence (β = 0.178, *p* = 0.165) had a significant indirect effect on the health behaviors of adolescents with moyamoya disease. Only the sense of coherence (β = 0.467, *p* < 0.001) had significant direct and total effects on subjective health status.

As a result of variables showing significant values for endogenous variables of quality of life, health behavior (β = −0.173, p = 0.009) had a standardized direct effect of 8.5% and a standardized total effect of 8.5%. Stress (β = −0.557, p < 0.001) had a significant, standardized direct effect and 40.0% standardized total effect on the quality of life of adolescents with moyamoya disease by 41.4%. Sense of coherence was a significant standardized direct effect of 50.4% (β = 0.371, p = 0.003) on quality of life, a standardized indirect effect of 29.9% (β = 0.220, p = 0.013) and a standardized total effect of 80.3% (β = 0.590, p < 0.001) affected. That is, consistency and stress were the most important factors in predicting quality of life (Table 4).

## Discussion

This study established the first health status model for adolescents with moyamoya disease based on the salutogenesis theory and is meaningful in that it examines their health behaviors and status. These health-related indicators include social support, sense of coherence, health behavior, and stress. The factor in this model that had the most significant influence on the health status of adolescents with moyamoya disease was stress. This supports the salutogenesis theory that sense of coherence and successful stress management influences an individual's health status^14,27^.

The stress score of adolescents with moyamoya disease measured in this study was 43.71 points, which was higher than the 42.50 points of the high-risk group of the middle and high school adolescent stress scale test. This score indicates that adolescents with moyamoya disease fall into the high-risk group for stress among South Korean adolescents^30^. Probable causes for the increased stress in this group include everyday limitations and psychological tension or pressure caused by the disease, and regular medical treatment and examination^3^. Further analysis indicates that the stress of the adolescents in this study was particularly high in the physical domain, followed by the emotional and behavioral domains. These results mirror those of a stress-related survey on adolescents with chronic diseases, in which physical discomfort and limitations due to health problems were some of the leading causes of stress^36^.

Sense of coherence had the most significant influence as a variable explaining the stress of adolescents with moyamoya disease. This supports the results of previous studies where adolescents with a high sense of coherence maintained good health and managed stressful situations well^14,16,20^. According to the salutogenesis theory, people with a high sense of coherence are more flexible and use adaptive strategies to cope with specific situations and to meet their needs^14^. The sense of coherence refers to managing personal tension, identifying internal and external resources, and mobilizing them to find solutions to effective stress management. A previous study on adolescents with chronic heart disease showed that the group with a high sense of coherence had a lower degree of exhaustion^37,38^. Furthermore, a report showed a negative correlation between the sense of coherence and stress in epileptic adolescents, which supports the results of this study^39^.

Our study found that sense of coherence directly affected subjective health status and had a significant indirect effect on health behavior. This supports the results of previous studies that show that sense of coherence had a significant correlation with health status^40,41^ and promoted health behaviors^42,43^.

The indirect effect of moyamoya disease on health behavior (treatment instructions, health coping behavior, health promotion acts) in this study indicates that, as in previous studies, sense of coherence helps with lifestyle modification^44^.

The structural model showed that social support had a significant direct effect on health behavior and lowered stress even more than sense of coherence. This supports the results of previous studies that reported positive coping and stress reduction through family and social support systems^45–47^. Moyamoya health behavior and stress directly affect the quality of life of adolescents with the disease.

This indicates that the quality of life of adolescents with chronic diseases is higher in groups with good individual health behaviors and the ability to cope with stress^48,49^. On the other hand, sense of coherence has both a direct and indirect effect on quality of life. Previous studies that applied the salutogenesis theory found that the higher the sense of coherence, the fewer stress symptoms there were and the better the individual's quality of life^50,51^. Higher sense of coherence was an indicator of better health behaviors^27^ and improved the quality of life. Therefore, sense of coherence is a significant factor affecting quality of life^14^. This result suggests that interventions for increasing sense of coherence in adolescents with moyamoya disease are critical. Therefore, effective strategies, such as developing of a program to increase the sense of coherence considering the characteristics of the patients (moyamoya disease, adolescents) should be considered to improve the quality of life of adolescents with moyamoya disease.

The model analysis in this study showed that sense of coherence is a predictor of stress, health behavior, subjective health status, and quality of life in adolescents with moyamoya disease. It can therefore be said to be a model supporting the salutogenesis theory. Health promotion based on the salutogenesis theory refers to a process in which adolescents with chronic diseases become the participants themselves, identify the causes of stress in their lives, and use appropriate resources to address them. It is necessary to provide health resources, social support, and interventions that enhance sense of coherence to improve the health of adolescents with moyamoya disease. As they are more likely to be stressed due to their affliction, it is necessary to consider developing a program to promote health and improve their sense of coherence.

Sense of coherence develops when the norms and values of life are apparent, when there are positive experiences of success, when people have access to social resources, and when they are respected in the decision-making process. Lack of social relationships, negative life experiences, and lack of education can hinder the development of sense of coherence^52,53^. Therefore, to improve the sense of coherence of adolescents with moyamoya disease, it is necessary to promote good health behaviors and activities, provide positive reinforcement when implementing them, and plan interventions that connect them to health provider networks.

Our study differs from previous studies because it first considered the quality of life of adolescent patients with moyamoya disease. In addition, through a literature review based on health-related theories, we found that certain variables (social support, sense of coherence, stress, health behavior implementation, health status factors) can affect the quality of life of adolescents with moyamoya disease. This is the first domestic and other national study to show this.

But several limitations should be considered when interpreting the results of this study. First, this study is based on a cross-sectional sample. Longitudinal samples are required to understand long-term observational studies on the sense of coherence and health status of adolescents with moyamoya disease. Second, this study was based on the adolescents' subjective interpretation of each item in the questionnaire. Lastly, since this study was conducted only on adolescents with moyamoya disease at a hospital in Seoul, there is a limit to generalizing the results to all adolescents with moyamoya disease.

## Conclusions

In this study, we analyzed the factors that affect the quality of life of adolescents with moyamoya disease and we established a structural model based on Antonovsky's salutogenesis theory to identify how various influencing factors.

According to this structural model analysis, sense of coherence was influencing factor in lowering stress, social support and stress were influencing factors implementation of health behavior in adolescents with moyamoya disease.

Sense of coherence and stress were major influencing factors in improving the health status and quality of life in adolescents with moyamoya disease.

Based on these results, developing and providing intervention programs to enhance sense of coherence and managing stress may decrease stress, which may be a great strategy for improving the quality of life of adolescents with moyamoya disease.

### Relevance to clinical practice

Since moyamoya disease, a chronic disease, is progressive, it is very important to identify factors for health promotion. The findings from this study have the potential to help nurses to promote the health of adolescents with moyamoya disease and other chronic diseases. Sense of coherence and social support were major influencing factors in lowering stress and improving the health status and quality of life in adolescents with moyamoya disease.

We recommend that nurses monitor the symptoms of adolescents with moyamoya disease, explore how the patients minimize their symptoms and how confident they feel about managing their symptoms—particularly stress and healthy behaviors.

Also, this paper is intended to help health experts to develop an intervention strategy based on theory as an approach for chronic disease management.

## Data Availability

The datasets used and/or analyzed during the current study are available from the corresponding author on reasonable request.
